# Errata

**Published:** 1867-01

**Authors:** 


### ERRATA.

In the article entitled Compensation, in the Dec. No. of the REGISTER, on page 534, third line from top, read "distribution" instead of "retribution."

And on page 535, 13th line from bottom, read "But" instead of "At." Also same page, 5th line from bottom, read "obedience" instead of "and obedience." Also on page 563, last line at bottom, for "few and many" read "pen and money." And on page 564, 11th line from top, for "runner" read "income."

